# The Future of Onchocerciasis Control in Africa

**DOI:** 10.1371/journal.pntd.0000074

**Published:** 2007-10-31

**Authors:** Catherine Hodgkin, David H. Molyneux, Adenike Abiose, Bernhard Philippon, Michael R. Reich, J. Hans Remme, Bjorn Thylefors, Mamadou Traore, Karen Grepin

**Affiliations:** 1 Royal Tropical Institute, Amsterdam, The Netherlands; 2 Lymphatic Filariasis Support Centre, Liverpool School of Tropical Medicine, Liverpool, United Kingdom; 3 Sightcare International, Ibadan, Oyo State, Nigeria; 4 Institut de Recherche pour le Développement, Paris, France; 5 Harvard School of Public Health, Boston, Massachusetts, United States of America; 6 Special Programme for Research and Training in Tropical Diseases, World Health Organization, Geneva, Switzerland; 7 Mectizan Donation Program, Task Force for Child Survival and Development, Atlanta, Georgia, United States of America; 8 Institut National de Recherche en Santé Publique, Bamako, Mali; 9 Harvard University, Boston, Massachusetts, United States of America; Swiss Tropical Institute, Switzerland

## Past Achievements in the Control of Onchocerciasis

**Figure 1:**
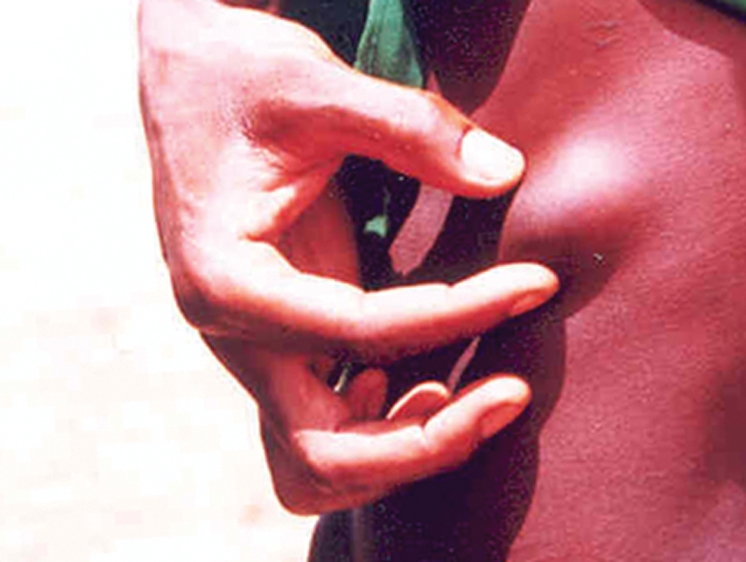
Palpable Onchocercal Nodule.

**Figure 2:**
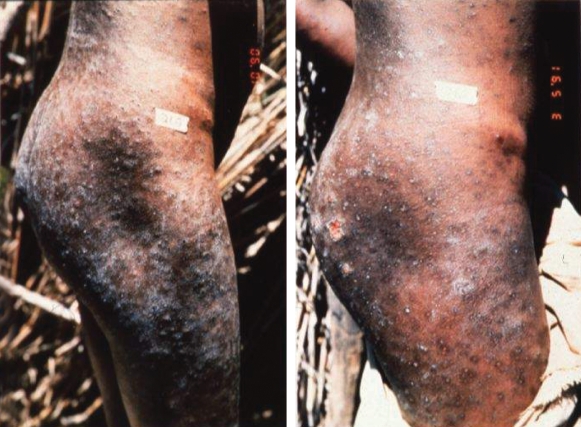
Onchocercal Skin Disease.

Large-scale control of onchocerciasis commenced over three decades ago, initially through the Onchocerciasis Control Programme in West Africa (OCP, 1974–2002), and more recently by the African Programme for Onchocerciasis Control (APOC, 1995–2010). The goals of OCP were to eliminate onchocerciasis as a public health problem and to mitigate its negative impact on the social and economic development of affected regions [1–3]. The strategic objective of APOC is to permanently protect the remaining 120 million people at risk of this debilitating and disfiguring disease in 19 countries in Africa through the establishment of community-directed treatment with ivermectin (CDTI) that is capable of being sustained by the communities after APOC financing has ended (Figures 1 and 2). The achievements made to date, first by OCP and subsequently by APOC, are summarised in Table 1.

**Table 1:** Achievements of OCP and APOC.

OCP Results (1974–2002)	APOC Results (1996–2005)
• Infection and eye lesions prevented in 40 million people in 11 countries	• 40 million people in 16 countries under regular ivermectin treatment
• 600,000 cases of blindness prevented	• 500,000 DALYs per year averted
• 25 million hectares of abandoned arable land reclaimed for settlement and agricultural production, capable of feeding 17 million people annually	• 177,000 communities mobilised
	• Workforce of 261,000 community distributors trained and available for other programmes
• Economic rate of return of 20%	• Economic rate of return of 17%
	• US$7 per DALY averted

DALY, disability-affected life year.

The long-term support of onchocerciasis control, together with sustained political commitment of national governments, bilateral donors, and non-governmental development organisations (NGDOs), is a major, yet unheralded public health and development success story in Africa. The regional approach and the emphasis on continued operational research were also critical factors in the success of the OCP. In addition, the contributions of OCP and APOC to health systems development, especially at the peripheral level, have been emphasised as an example of how disease programmes can strengthen resource-poor health systems [3,4]. The contribution of a diverse group of stakeholders to the success of the OCP and APOC partnerships has recently been reviewed [5]. In APOC, the collaboration between NGDOs and the Ministries of Health (through designated national onchocerciasis task forces [NOTFs]) is contributing some 25% of resources to onchocerciasis control, assisting in national capacity building and in the implementation of APOC projects. The national task forces will continue to fulfil these functions and obligations at the same level of commitment up to at least 2015.

Clearly, OCP and APOC have demonstrated that inter-country collaboration can tackle a major public health problem. Both programmes have secured long-term funding for ivermectin treatment through a public–private partnership, and have succeeded in scaling up interventions by gaining country commitment and mobilising community involvement [3]. Control efforts have had a significant impact on both public health and development objectives in affected regions [2–5]. For example, investments in onchocerciasis control have one of the highest economic rates of return among international development initiatives: an estimated 15%–20% (see Box 1). In addition, the successes of OCP increased accessibility to fertile land, which in turn enhanced agricultural yields, led to elevated crop diversity, improved human nutritional status, and removed two significant public health problems, namely onchocercal blindness and skin disease [6]. Whilst the OCP itself ended in 2002, activities continued in special intervention zones (SIZ) where there were unsatisfactory epidemiological situations suggesting the potential for recrudescence and results in 2002 that were not compatible with the achievement of the OCP objective. Areas in Benin, Togo, Ghana, Guinea, and Sierra Leone were selected as special intervention zones. The major strategy involved delivery of ivermectin, but in Togo and Benin aerial larviciding was also employed.

**Box 1. Strategic Recommendations**

**1. APOC's main objective of establishing sustainable national onchocerciasis programmes in all countries where needed should be maintained and endorsed.**

**2. APOC's operations should be extended to 2015 to enable it to fulfil its original objectives.**

• APOC should complete the establishment of CDTI projects in all countries.

• APOC should develop an exit strategy that includes effective long-term support mechanisms for countries to provide the limited support to CDTI and onchocerciasis surveillance necessary after the programme has come to an end.

**3. APOC should develop the evidence base to determine when and where ivermectin treatment can be stopped, and provide guidance to countries on how to prepare for and evaluate cessation of treatment.**

• The group emphasised that there is insufficient knowledge about when and how mass treatment can be stopped.

• The group suggested that APOC develop a plan, including resource requirements, to monitor progress in areas where cessation of ivermectin treatment has occurred.

**4. APOC should promote integration and co-implementation of interventions with CDTI to provide multiple health benefits to large populations.**

• APOC should advocate for integration of CDTI into strengthened health systems, and develop plans for facilitation, advocacy, and exchange of best practices.

• APOC should encourage joint financing and advocacy for NTD partnerships.

• Partners should continue to strengthen operational research on co-implementation, and NTD partnership representatives should participate in the Joint Action Forum to promote harmonisation of partnerships.

**5. APOC's mandate should be extended to include all onchocerciasis-endemic countries in Africa where the epidemiological situation requires sustainable CDTI.**

• This will ensure continuity of support to special intervention zones and former OCP countries where sustainable CDTI still needs to be established.

• The capacity of the Multi-Disease Surveillance Centre to fulfil its anticipated role in relation to onchocerciasis surveillance should be strengthened.

**6. Financial planning and fundraising for onchocerciasis control should build on existing mechanisms and traditional donors, but should also explore new funding opportunities, particularly those offered in the context of NTDs.**

• Financial planning and fundraising will need to take into account the need to secure country commitments to stable funding to achieve sustainable country programmes.

• Financial planning and fundraising should cover the essential activities of the extension to 2015 to allow APOC to fulfil its core mandate and the additional activities identified in the strategic overview.

• Plans for an exit strategy should include the costing of core regional functions that will be necessary post-APOC, together with an overview of which partners and actors may be able to take on these functions and the necessary resource requirements.

## From Vector Control to Mass Drug Administration via Community-Directed Treatment with Ivermectin

Vector control using aerial application of larvicides was the initial strategy of the OCP, with the aim of interrupting onchocerciasis transmission. It was hypothesised that maintaining this control strategy for 14 years (initially it was considered that 20 years was necessary) would result in adult onchocercal parasites dying out naturally in the human hosts. This strategy was successful in the central OCP area (∼1 million km^2^) where onchocerciasis infection and transmission were eliminated, and where active control could cease and be replaced by surveillance [2]. However, in the APOC countries, vector control was considered neither feasible nor cost-effective, except for a few small isolated foci in East and Central Africa [7,8] where criteria were established for financing of vector control. The underlying rationale was that the foci were isolated, and hence there was no risk of reinvasion of adjacent blackfly populations. These foci were in Uganda (Itwara), Tanzania (Tukuyu), and on Bioko island in Equatorial Guinea [7].

The current mainstay for the control of onchocerciasis rests on mass drug administration (MDA) using ivermectin (brand name Mectizan). Ivermectin was registered in 1987 and subsequently donated to affected communities by Merck & Co. Inc., providing a drug for disease intervention that was applicable in all endemic areas [9]. Ivermectin distribution was first introduced in ex-OCP countries [10]. Ivermectin is effective against the microfilariae that cause the severe manifestations of the disease, and MDA of ivermectin proved an effective strategy for eliminating onchocerciasis as a public health problem. The main limitation of ivermectin is that it has little effect on the adult worms that continue to produce microfilariae, and hence re-treatment is required at annual intervals. MDA of ivermectin reduces but does not interrupt transmission, at least not during the first years of intervention, and annual treatment needs to be continued for a long period of time. In other words, APOC's objective was to establish “effective and sustainable community-directed treatment with ivermectin (CDTI) in all endemic areas” [5,11].

The necessary duration of ivermectin treatment has yet to be determined, and likely depends on treatment coverage and initial levels of endemicity. Two scenarios emerge, namely (1) continued treatment in the most endemic foci where transmission cannot be interrupted, and (2) cessation of treatment in less affected areas after prolonged treatment. Although the second scenario is as yet hypothetical, it is currently under evaluation to test its feasibility.

The overarching goal of APOC is to establish sustainable CDTI in all countries where onchocerciasis remains endemic. APOC's achievements have been summarised before [7,8], but there have been delays in implementation of CDTI projects [3] due to problems in conflict and post-conflict areas and the treatment complications linked to onchocerciasis and loasis co-endemicity. In areas of *Loa loa* there is a risk of severe adverse events in people treated with ivermectin who have a high microfilaraemia of *L. loa,* which can result in encephalopathy [12,13]. This has been a major impediment to the expansion of APOC in Central Africa. However, it is expected that by 2007 CDTI projects will have been launched in all areas in Africa where onchocerciasis is, or has been, a public health problem.

## Working Group on the Future of Onchocerciasis Control in Africa

Against the background outlined above, and in the light of an external evaluation of APOC in 2005, the Committee of Sponsoring Agencies established a working group in 2006, at the request of the governing body of APOC at the 11th meeting of the Joint Action Forum in Paris. The working group was given a mandate to reflect on past achievements and forthcoming challenges and opportunities of APOC and its partners, and to put forward recommendations on the future of onchocerciasis control in Africa. The full report from the working group is available as Supporting Information File Text S1. The following points were emphasised.

First, through CDTI, APOC has worked to eliminate onchocerciasis as a public health problem, an effort that has also mitigated the disease's impact on socio-economic development in affected regions. In view of APOC's success in onchocerciasis control, any premature closure of the programme would lead to the loss of the many benefits derived and to the deterioration of the CDTI infrastructure, which could also serve as an effective platform for the delivery of other health interventions that are needed by many millions of the under-served and poorest people in Africa, and that address the millennium development goals.

Second, the group saw three main challenges for onchocerciasis control: (1) how can an adequate treatment coverage with ivermectin be established and sustained in those African settings where MDA is indicated?; (2) what are the means to determine where and when treatment can be stopped?; and (3) how does one ensure effective surveillance in areas where active control has come to an end?

Third, Africa needs to maintain the gains made in controlling onchocerciasis by both the OCP and APOC and to continue to reduce the impact of the disease as a pressing public health problem and an impediment to social and economic advances. Much is being achieved by sustainable country programmes, and hence it is necessary to ensure long-term sustainability through the following approaches: (1) Continued advocacy to maintain commitment to onchocerciasis control; (2) Monitoring and evaluation of control programmes and support to post-control surveillance; (3) Maintenance of a core body of onchocerciasis experts to provide a forum to exchange expertise and experiences; and (4) Long-term financial sustainability of onchocerciasis control.

The working group's vision of APOC is of an effective organisation that lacks strict geographical boundaries and can address the need for advocacy, technical assistance, and continued support for onchocerciasis control throughout endemic areas of Africa. The group suggested that APOC should promote innovation at the community level and ensure the sustainability of onchocerciasis programmes through its operational philosophy of CDTI, as well as harnessing additional resources to assist countries in co-implementation of other health interventions where appropriate.

Control efforts will require the further development and refinement of a flexible approach where countries can call on different levels of support from APOC according to their capacity and accomplishments to date. We are not suggesting that a regional onchocerciasis control programme with an indefinite end point be established. However, continued success requires long-term coordinated support, continuity of that support, and a careful definition of the roles and responsibilities of different actors and stakeholders.

A number of recommendations emanated from the review process and these are summarised in Box 1. Additional recommendations were also put forward as implicit or essential to achieve the strategic recommendations. The group focused on selected issues relating to the future of onchocerciasis control in Africa.

## Conclusion

Onchocerciasis control in Africa since 1974 has been one of the most successful health and development activities in terms of public health achievement, partnership development, sustained donor support, and social and economic development. The group reviewed the complex issues related to onchocerciasis control, identifying those which must be addressed over the next decade. The landscape of international health has changed much since the inception of APOC in 1995. In recent years, however, there has been a renewed interest in and enhanced profile of neglected tropical diseases (NTDs), and the success of onchocerciasis control is recognised as a contributing factor, as it is a cost-effective intervention that addresses the millennium development goals and builds partnerships with stakeholders in the international health community. Onchocerciasis control would be greatly assisted if there were a drug that killed adult worms (a macrofilaricide), and research to this end must continue.

APOC, the group concluded, needs to be provided with the resources through its traditional donor base to continue until 2015, not only using its CDTI approach to achieve control of onchocerciasis, but building on this strategy to deliver other health interventions and scale up NTD control. The challenge is to translate the country commitment as expressed in the Yaoundé Ministerial Declaration [14] towards a sustainable and integrated approach for surveillance and control within strengthened health systems. As with other disabling infections, onchocerciasis is not always seen as a priority and must compete with other higher profile diseases for resources. However, its control is regarded as one of the most cost-effective and successful health and development partnerships of recent decades [15]. Further information on recent progress is summarised in the World Health Organization's Weekly Epidemiological Record [16].

## Supporting Information

Text S1 Full Report from the APOC Working Group
(0.67 MB PDF)
