# Supplementary material for: The Future of Onchocerciasis Control in Africa
Source: PLoS Negl Trop Dis. 2007 Oct 31;1(1):e74. doi: 10.1371/journal.pntd.0000074 (PMC2041822; doi:10.1371/journal.pntd.0000074)
Supplement: Text S1 — Full Report from the APOC Working Group (0.67 MB PDF) [file pntd.0000074.s001.pdf]

COMMITTEE OF SPONSORING AGENCIES
Office of the Chairman

PARTNERS' MEETING ON ONCHOCERCIASIS CONTROL IN AFRICA
Yaoundé, 26-27 September, 2006

APOC

African Programme for Onchocerciasis Control
Programme africain de lutte contre l'onchocercose

A STRATEGIC OVERVIEW OF THE FUTURE OF ONCHOCERCIASIS CONTROL IN AFRICA

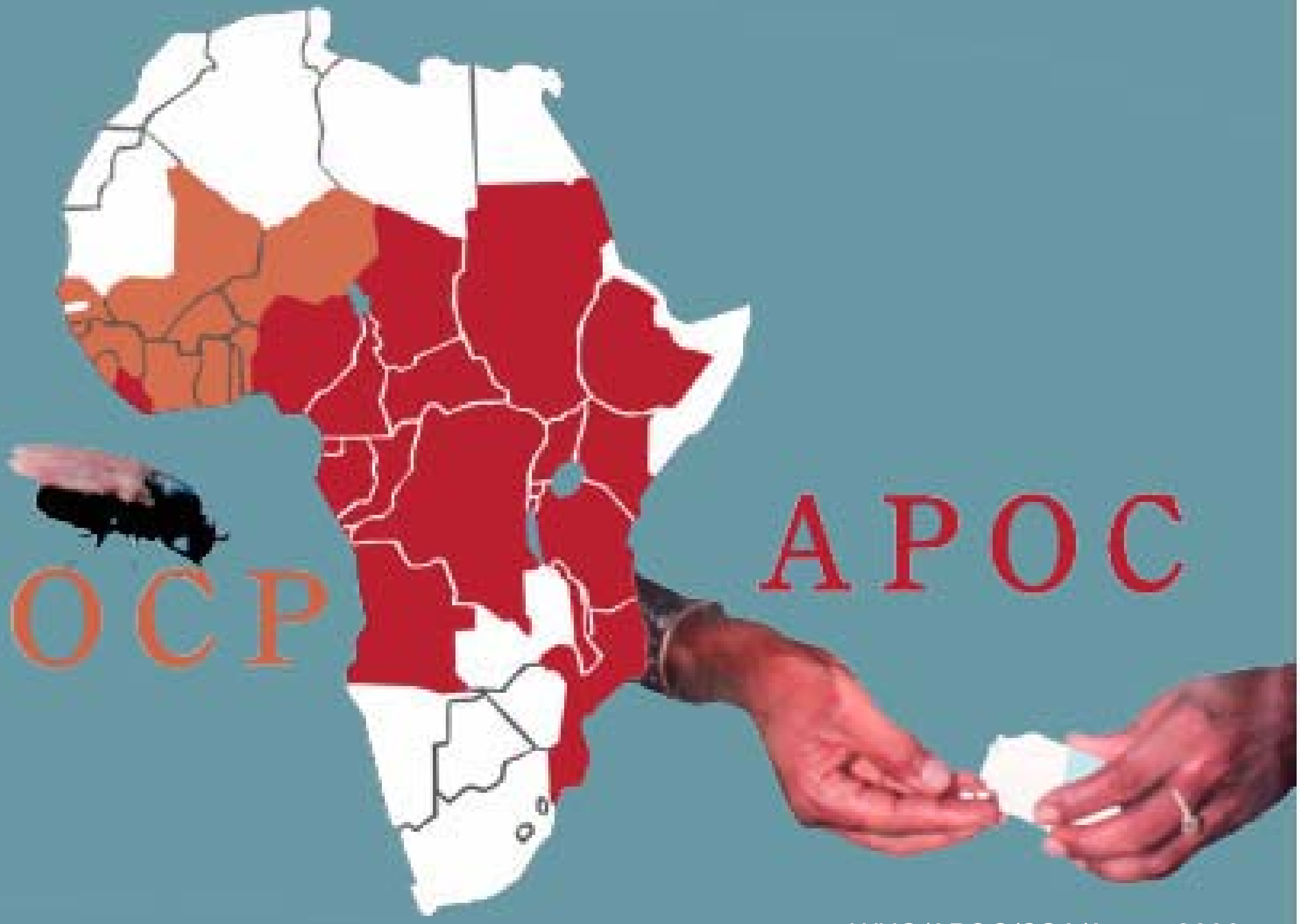

WHO/APOC/CSA/August2006
ORIGINAL: ENGLISH

TABLE OF CONTENTS

ACRONYMS II

EXECUTIVE SUMMARY III

INTRODUCTION 1

ONCHOCERCIASIS CONTROL STRATEGIES, ACHIEVEMENTS, AND FUTURE CHALLENGES . 3

A CHANGING CONTEXT FOR ONCHOCERCIASIS CONTROL 8

ISSUES FOR CONSIDERATION 10

WORKING GROUP MEMBERS 25

ANNEX 1: TERMS OF REFERENCE 26

ANNEX 2: LIST OF CONSULTATIVE MEETING PARTICIPANTS 31

ANNEX 3: LIST OF KEY DOCUMENTS 33

The Working Group wishes to express its appreciation of the support given to the Group by APOC management and staff who have willingly provided documentation, information and support to the meetings of the Group. In consultative meetings in Ouagadougou, all meeting participants were rigorous and innovative in discussions about the future of onchocerciasis control. We appreciate the positive input and the generosity with which people have shared their time and ideas with us.

ACRONYMS

AFRO	African Regional Office of WHO
APOC	African Programme for Onchocerciasis Control
CDTI	Community Directed Treatment with Ivermectin
CDD	Community Directed Distributor
CSA	Committee of Sponsoring Agencies
HIPC	Heavily Indebted Poor Countries Initiative
JAF	Joint Action Forum
LF	Lymphatic Filariasis
MDGs	Millennium Development Goals
MDP	Mectizan Donation Programme
MDSC	Multi-Disease Surveillance Center
MoH	Ministry of Health
MTEF	Medium Term Expenditure Framework
NGDO	Non-Governmental Development Organization
NOCP	National Onchocerciasis Control Programme
NOTF	National Onchocerciasis Task Force
NTD	Neglected Tropical Disease
OCP	Onchocerciasis Control Programme in West Africa
PEPFAR	The President's (US President's) Emergency Plan for AIDS Relief
SAEs	Severe Adverse Effects
SIZ	Special Intervention Zone
TCC	Technical Consultative Committee (of APOC)
RAPLOA	Rapid Assessment Procedure for Loiasis
REMO	Rapid Epidemiological Mapping of Onchocerciasis
TDR	UNICEF/UNDP/World Bank/WHO Special Programme for Research and Training in Tropical Diseases
WHO	World Health Organization

EXECUTIVE SUMMARY

This paper summarizes the reflections and recommendations of the Working Group (hereafter the Group) on the Future of Onchocerciasis Control in Africa. The Group has reflected in depth on the challenges to and options for onchocerciasis control, and on the future role of the African Programme for Onchocerciasis Control (APOC) and its partners.

Through Community Directed Treatment with Ivermectin (CDTI), APOC has worked to eliminate this debilitating and disfiguring disease as a public health problem, an effort that has also mitigated the disease's impact on socio-economic development in affected regions. APOC has achieved many significant successes in onchocerciasis control, and premature closure of the programme would lead to the loss of the many benefits derived from the activities of the last several years and to the deterioration of the CDTI infrastructure which could serve as an effective platform for the delivery of other health interventions needed by millions of under-served people in Africa and which address the Millennium Development Goals (MDGs).

The Group sees the future challenges for onchocerciasis control as follows: (i) to establish and sustain adequate treatment with ivermectin in all areas in Africa where mass treatment with ivermectin treatment is indicated[1], (ii) to determine where and when treatment can be stopped, and (iii) to ensure effective surveillance in areas where active control has come to an end. In addition, Africa needs to maintain the gains made in controlling onchocerciasis by the Onchocerciasis Control Programme (OCP) and APOC and to continue to reduce the impact of the disease on public health. Much is being achieved by sustainable country programmes but it is necessary to ensure in the long-term:

1. Continued advocacy to maintain commitment to onchocerciasis control;
2. Monitoring and evaluation of control programmes and support to post-control surveillance;
3. Maintenance of a core body of onchocerciasis expertise to provide a forum to exchange knowledge and ideas;
4. Long-term financial sustainability of onchocerciasis control.

The Group's future vision of APOC is of an effective organization that lacks strict geographical boundaries and can address the need for advocacy, technical assistance and continued support for onchocerciasis control throughout the entire African region. A programme which continues to promote innovation at the community-level and promotes the sustainability of onchocerciasis programmes using its core operational philosophy of CDTI to harness additional resources and assist countries in co-implementation of other health interventions where appropriate.

Control efforts will require the development of a flexible approach in which countries can call on different levels of support from APOC according to their capacity and the level of progress accomplished to date. The challenges and the need for on-going activities does not imply a regional onchocerciasis control programme with an indefinite end point; however, continued success will require long-term coordinated support, continuity of that support and a careful definition of the roles and responsibilities of different actors.

[1] Areas meso- or hyper-endemic for onchocerciasis.

The key strategic recommendations of the group are given in bold. A number of additional recommendations are also put forward as implicit or essential to achieve the strategic recommendations. The Group sought not to repeat or prioritize the many recommendations of the recent External Evaluation, which has been accepted by the Joint Action Forum (JAF), but focused on selected key issues relating to the future of onchocerciasis control in Africa.

The Group recommends that:

Recommendation 1: APOC's main objective of establishing sustainable national onchocerciasis programmes in all countries where needed should be maintained and endorsed.

Recommendation 2: APOC operations should be extended to 2015 to enable it to fulfill its original objectives.

- APOC should complete the establishment of CDTI projects in all countries;
- APOC should develop an exit strategy which includes effective long-term support mechanisms for countries including mechanisms to provide the limited support to CDTI and onchocerciasis surveillance that will remain necessary after the programme has come to an end.

Recommendation 3: APOC should take on the additional objective of developing the evidence base to determine when and where ivermectin treatment can be stopped, and provide guidance to countries on how to prepare for and evaluate cessation of treatment.

- The JAF is asked to endorse this new objective recognizing that at present there is insufficient knowledge about when and how mass treatment can be stopped;
- APOC should develop a plan, including the excluding resource requirements, to monitor progress in selected areas where cessation of ivermectin treatment has occurred.

Recommendation 4: APOC should promote integration and co-implementation of interventions with CDTI to effectively provide multiple health benefits to large populations.

- APOC should advocate for integration of CDTI into strengthened health systems;
- APOC should develop plans for facilitation, advocacy, and exchange of best practices;
- The CSA and the APOC Partnership should encourage joint financing and advocacy for Neglected Tropical Disease (NTD) partnerships;
- APOC, TDR, and partners should continue and strengthen operational research on co-implementation;
- The CSA should invite other NTD partnership representatives to JAF to promote harmonization of partnerships.

Recommendation 5: APOC's mandate should be extended to include all onchocerciasis endemic countries in Africa where the epidemiological situation requires sustainable CDTI.

- The JAF is recommended to endorse this extended mandate which can be executed within the terms of the existing Memorandum;
- APOC will thus ensure continuity of support to Special Intervention Zones (SIZ) and former OCP countries where sustainable CDTI still needs to be established;
- APOC partners should, promote actions to strengthen the capacity of the MDSC to fulfill its anticipated role in relation to onchocerciasis surveillance.

Recommendation 6: Financial planning and fundraising for onchocerciasis control should build on existing mechanisms and traditional donors but should also explore new funding opportunities particularly those offered in the context of NTDs.

- Financial planning and fundraising will need to take into account the need to secure country commitments to stable funding for sustainable country programmes;
- Financial planning and fundraising should cover the essential activities of the extension to 2015 to allow APOC to fulfill its core mandate and the additional activities identified in the strategic overview;
- Planning for an exit strategy should include costing of core regional functions that will be necessary post-APOC together with an overview of which partners and actors may be able to take on these functions and the necessary resource requirements.

INTRODUCTION

This paper summarizes the reflections and recommendations of the Working Group on the Future of Onchocerciasis Control in Africa. The Group was commissioned by the Committee of Sponsoring Agencies (CSA) following a recommendation made during the Joint Action Forum (JAF), in response to the recommendations of the 2005 External Evaluation and the 2005 Mid-Term Evaluation of the Special Intervention Zones (SIZ).[2]

As recommended by JAF11 the Group was asked to reflect on the following points:
- a) *The future of the Programme until 2010 including the modalities of an eventual extension;*
- b) *Modalities of continuing control activities in the countries (transfer, integration, funding and advocacy);*
- c) *Geographical extension: APOC, SIZ areas (after 2007), ex-OCP countries and countries in conflict situation;*
- d) *Eventual financing before and after 2010.*

The Group identified and considered four specific and not mutually exclusive issues facing APOC and onchocerciasis control, reflected upon the implications of these issues, and has made recommendations on the future of APOC and onchocerciasis control in Africa. The four issues at the core of discussions by the Group were whether:

1. To extend APOC duration and strengthen its capacity;
2. To integrate CDTI with additional health interventions;
3. To broaden the mandate of APOC to include all onchocerciasis endemic countries in Africa;[3]
4. To suggest strategies for addressing future financial needs.

During the spring of 2006, the Group met on three occasions and discussed the future of APOC with numerous programme members and partners. In addition to these activities, the Group consulted with other organizations as they saw appropriate. This paper is the result of this process. It summarizes the methods used by the Group in their deliberations, provides an analysis of the implications surrounding the four core issues addressed by the Group, and provides conclusions and recommendations based upon these discussions.

---

[2] See Appendix 1 for Terms of Reference (ToR) of the Working Group.

[3] In the Group's ToR, the wording is "to include former OCP countries." The Group suggested referring to all onchocerciasis endemic countries in Africa meaning all countries where mass treatment is indicated. The Group is not suggesting a role for APOC in countries where this is not needed. The Group also suggested that APOC extend some level of technical support to Yemen on request to achieve an integrated programme.

METHODS

The Group convened for the first time immediately following a Consultative Meeting held on March 20th and 21st, 2006, in Ouagadougou, Burkina Faso. The Consultative Meeting brought together representatives of APOC countries, Non-Governmental Development Organizations (NGDOs), and donors for discussions about their experiences and views.[4] During this meeting, the Group had an opportunity to discuss the main discussion points of the Consultative Meeting, findings of the 2005 External Evaluation, the 2005 Mid-Term Evaluation of the Special Intervention Zones (SIZ), and other documents from APOC and APOC partners.[5] A preliminary agenda and plan for the development of the strategic overview were established at this initial meeting. The Group also convened for a three day working session in Geneva and a final meeting in Paris. In addition to meetings of the Group, a virtual discussion forum was established in which documents could be shared and reviewed via the World Wide Web.

The Group used the recent evaluations and documents as a starting point for discussion. The goal was to synthesize findings and key issues, discuss their implications, and strategize for the future. The Group acknowledged that useful analysis had been completed thus far in identifying the most pressing issues facing APOC. Its members came to a consensus that the four major points for discussion regarding the future of APOC were identified in the Terms of Reference, and looked to the evaluation and other documents to help elucidate the implications of these core issues.

These sessions produced a rich discussion of the issues facing APOC, including the implications and potential solutions. The Group used many of the points from the Consultative Meeting in the development of this report's structure and analysis.

[4] See Appendix 2 for a full list of the Consultative Meeting participants.
[5] A full list of key documents considered by the Group is provided in Appendix 3.

ONCHOCERCIASIS CONTROL STRATEGIES, ACHIEVEMENTS, AND FUTURE CHALLENGES

Achievements

Control of onchocerciasis, coordinated first through the Onchocerciasis Control Programme in West Africa (OCP, 1974-2002) and more recently by the African Programme for Onchocerciasis Control (APOC, 1995-2010), has worked to eliminate this debilitating and disfiguring disease as a public health problem, and mitigated its impact on socio-economic development in affected regions. APOC, by building upon past successes, aims to permanently protect the remaining 120 million people at risk in 19 countries in Africa from the threat of onchocerciasis.[6] A number of the most important achievements of OCP and APOC are summarized in Table 1.

The long-term commitment to onchocerciasis control, and the maintenance of success through national governments and NGDOs is one of the major yet unheralded public health and socio-economic successes in Africa. A group of committed NGDOs has contributed very significantly to the OCP and APOC partnerships. These NGDOs work closely with the Ministries of Health through National Task Forces for Onchocerciasis Control (NOTFs) by contributing almost 25% of resources to APOC and by assisting in national capacity building and the implementation of APOC projects. It is expected that the NOTFs will to continue to fulfill these functions at the same level of commitment up to at least 2015 and possibly beyond.

OCP and APOC have demonstrated that inter-country collaboration can tackle a major public health problem. Both programmes have secured long term funding for ivermectin treatment through public private partnerships and have succeeded in scaling-up interventions by gaining country commitment and mobilizing community involvement.

Table 1: Achievements of OCP and APOC

OCP Results (1974-2002)	APOC Results (1996-2005)
• 40 million people in 11 countries prevented from infection and eye lesions	• 40 million people in 16 countries under regular ivermectin treatment
• 600,000 cases of blindness prevented	• 500,000 DALYs per year averted
• 25 million hectares of abandoned arable land reclaimed for settlement and agricultural production, capable of feeding 17 million people annually	• 117,000 communities mobilized<br>• Workforce of 261,000 community-directed distributors trained and available for other programmes
• Economic Rate of Return of 20%	• Economic Rate of Return of 17%<br>• US $ 7 per DALY averted

Control efforts to date have had a significant impact on both public health and development objectives in affected regions. Investments in onchocerciasis control have one of the highest economic rates of return, between 15 to 20%, among international development initiatives. The OCP increased accessibility to fertile land and hence increased agricultural yields, led to

[6] Meso- or hyper-endemic onchocerciasis areas where mass treatment with ivermectin is indicated.

crop diversity, and improved nutrition as well as removing the major public health problem of onchocercal blindness and skin disease.

Onchocerciasis Control Strategies

The initial strategy of the OCP was to use vector control to interrupt onchocerciasis transmission and maintain this control for 14 years until the adult onchocercal parasites in the human hosts died out naturally. This strategy was successful in the Central OCP area (1 million km^2) where onchocerciasis infection and transmission were eliminated, and where active control could be stopped and replaced by surveillance. In the APOC countries, vector control was not considered feasible or cost-effective, except for a few small isolated onchocerciasis foci in East and Central Africa.

The main control strategy for onchocerciasis is currently based on mass treatment with ivermectin (Mectizan®). The registration of ivermectin in 1987, and its subsequent donation by Merck & Co., Inc., provided a drug for disease intervention that was applicable in all endemic areas. Ivermectin distribution was first introduced in ex-OCP countries. Ivermectin is effective against the microfilariae that cause the severe manifestations of the disease, and mass treatment with ivermectin is an effective strategy for eliminating onchocerciasis as a public health problem. The main limitation of the drug is that it has little effect on the adult worms that continue to produce microfilariae, and re-treatment is required at annual intervals. Mass treatment with ivermectin reduces but does not interrupt transmission, at least not during the first years of intervention, and annual treatment needs to be continued for a long period of time. Hence APOC's objective is to establish "effective and sustainable community-directed ivermectin treatment in all endemic areas".

Currently data from impact assessment surveys is being analysed but the results were not yet available to the Group. The External Evaluation made recommendations regarding the introduction of impact assessment protocols and the expansion of impact assessment to all participating countries. The Group recognized that whilst the primary objective of APOC is to establish sustainable CDTI programmes; data on impact is important in priority setting.

How long ivermectin treatment needs to continue is not yet known but will depend on treatment coverage and initial level of endemicity. Two scenarios are likely: (1) continued treatment in the most endemic foci where transmission cannot be interrupted, and (2) cessation of treatment in less affected areas after prolonged treatment. Scenario 2 is as yet hypothetical but is under ongoing evaluation to test its feasibility. A schematic presentation of these two scenarios and the main stages of onchocerciasis control is given in Figure 1, and a more detailed description of the four control stages is given in Table 2.

Future Challenges

Onchocerciasis control will face three major challenges in the coming years: (i) to establish and sustain adequate treatment coverage with ivermectin in all areas in Africa where mass treatment with ivermectin is indicated, (ii) to determine where and when treatment can be stopped, and (iii) to ensure effective surveillance in areas where active control has come to an end.

The core objective of APOC focuses on the first challenge of establishing sustainable CDTI in all endemic countries. APOC has made remarkable progress but due to problems in

conflict areas and the treatment complications linked to onchocerciasis and loasis co-endemicity, another filarial disease in Central Africa, there have been delays. However, it is expected that by 2007 CDTI projects will have been launched in all areas in Africa where onchocerciasis is, or has been, a public health problem.

Figure 1: Possible Scenarios for Onchocerciasis Control

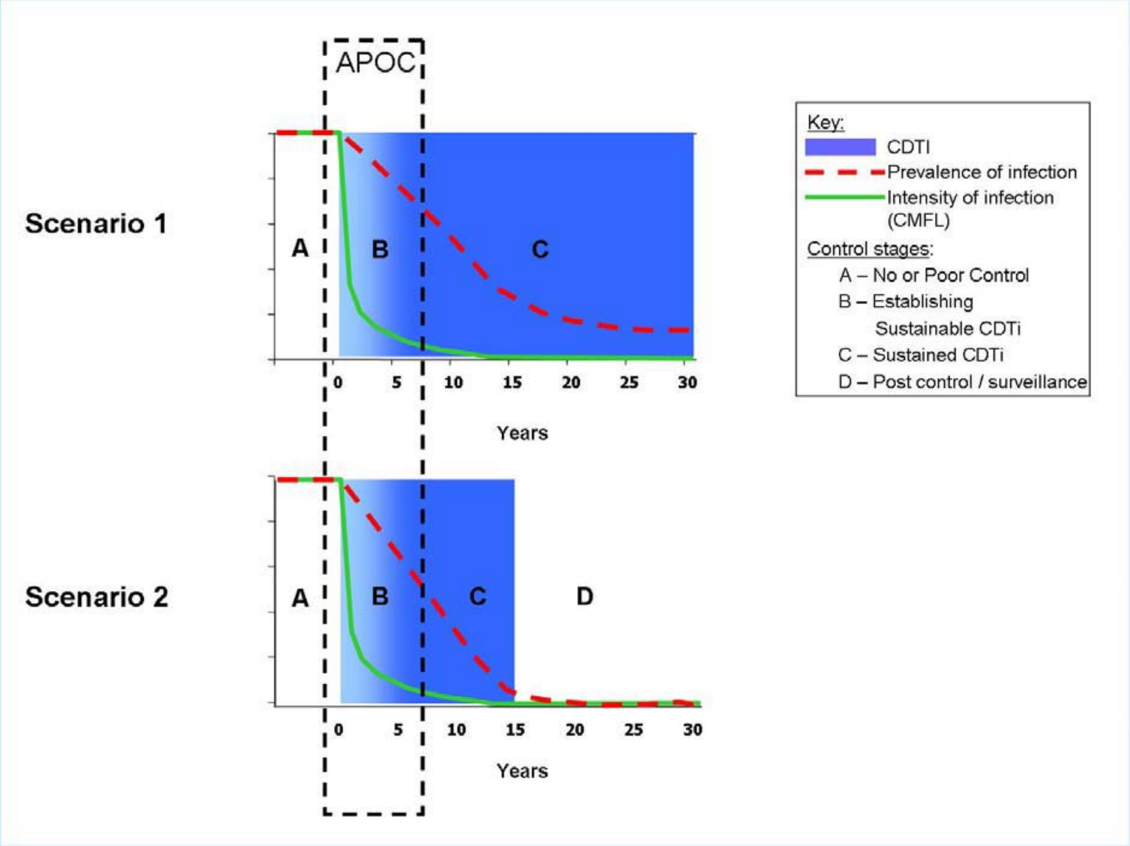

Note: In both scenarios the intensity of infection, and therefore the risk of disease, is falling rapidly to such low levels that onchocerciasis is no longer a public health problem. The prevalence of infection also declines but much slower. In scenario 1, the prevalence remains too high to interrupt transmission and annual treatment needs to be maintained. In scenario 2, the prevalence is reduced to near zero, transmission is interrupted and treatment can be safely stopped.

Preliminary data from a study in foci in Mali and Senegal, former OCP countries where treatment has been given for 18 years, suggests that onchocerciasis infection has been virtually eliminated, and the study will now proceed with testing whether ivermectin treatment can safely be stopped (Scenario 2). If this is the case we will have a new paradigm for onchocerciasis control in which elimination of transmission can be achieved through long-term ivermectin treatment. This example emphasizes the need for ongoing operational research for onchocerciasis control. There will be a need to determine in which areas this elimination will be feasible and to develop criteria and guidelines for stopping treatment in such areas. APOC will have to coordinate this process and support the necessary surveys, and this will be an important new objective for the Programme.

Whether transmission can be locally eliminated or not, onchocerciasis cannot be eradicated with ivermectin treatment and there will be a need for some limited but long-term support in all onchocerciasis areas after APOC support has come to an end and the programme has

closed.[7] The type of support that will be needed is defined in Table 2 for areas where CDTI needs to be sustained and for areas where transmission has been interrupted and treatment stopped, and where only surveillance will be needed. Such support is beyond the remit of APOC but it will be important to put the required mechanisms and arrangements into place before APOC closes to ensure that long-term low-level support continues in order to guarantee that the achievements of onchocerciasis control are maintained.

Research is ongoing to develop a drug that kills or permanently sterilizes the adult onchocercal worm. The availability of such a drug would greatly enhance the feasibility of achieving sustained elimination of onchocerciasis transmission. The most advanced candidate drug is moxidectin which is now entering phase II trials. The final step in the development plan for moxidectin is community trials to test whether it is more effective than ivermectin in reducing transmission. The results are expected by 2011 and if positive, moxidectin could be available for onchocerciasis control by 2012. Other promising candidate drugs are being investigated but are unlikely to be available for control before 2015 and there is no guarantee that any new product would be donated. There is also an urgent need for field applicable surveillance tools. The DEC patch test is still not operational and pool screening of black flies for infectivity using DNA techniques still faces operational challenges.

The scenarios in Figure 1 can also be used to classify onchocerciasis control in former OCP countries. While several former OCP countries are still free of onchocerciasis and in the 'post-control' stage of onchocerciasis control, Sierra Leone is now back in the first stage with virtually no control, and is in a comparable situation to post-conflict countries in APOC such as Angola, the Democratic Republic of the Congo (DRC), and South Sudan. In considering the future of onchocerciasis control in Africa, it would therefore be more useful to consider the stage of control in different areas rather than whether they belong to APOC or former OCP regions.

The achievements of both OCP and APOC have been remarkable but with the closure of OCP in 2002 and the planned closure of APOC in 2010 onchocerciasis control is entering a crucial stage and every effort must be made to ensure that the investments and successes of the past are sustained for the future. A critical examination of the current position and the changing context for onchocerciasis control is necessary to guide decisions about the future.

---

[7] Dadzie, et al. (2003) “Final report of the Conference on the eradicability of Onchocerciasis”. *Filaria Journal*. 2(2).

Table 2: Main Stages in Onchocerciasis Control

Stage of Control	Public Health problem	Characteristics	Location	Key issues	Support needed
A. Poor or no control	No change – morbidity due to blindness and skin disease remains high	• No CDTI or poor treatment coverage<br>• Unsatisfactory progress towards sustainability	• Conflict zones, e.g. Sierra Leone, Sudan Angola, Côte d"Ivoire<br>• Poorly performing projects in various APOC and former OCP countries<br>• Areas with high loiasis endemicity	• Control in conflict zones<br>• Overcome reasons for poor performance<br>• Treatment in areas with high loiasis endemicity	• Guidance and funding for developing sustainable CDTI<br>• Guidance for CDTI in conflict zones<br>• Guidance for CDTI in loiasis areas
B. Establishing sustainable CDTI	Declining	• CDTI being established with APOC funding<br>• Satisfactory treatment coverage	• Well performing APOC projects in their first 5-8 years<br>• SIZ areas in Guinea, Togo, and Bénin	• Establishing sustainable CDTI<br>• Integration with other community based interventions	• Guidance and funding for developing sustainable CDTI<br>• Guidance on integration<br>• Evaluation and sharing of experiences with CDTI and integration<br>• Monitoring progress
C. Sustained CDTI	Controlled/ Eliminated	• CDTI without OCP/APOC funding<br>• Sustained high treatment coverage	• Early APOC projects<br>• OCP: West / S.East Extensions (Senegal, Mali)	• Sustaining coverage for decades<br>• If, where, when and how to stop treatment	• Criteria and guidance for stopping CDTI<br>• Evaluation and sharing of experiences on sustaining CDTI
D. Post-control	Eliminated	• Transmission interrupted<br>• No active control, surveillance only	• Central OCP area<br>• Isolated foci in Uganda	• Sustained surveillance<br>• Recrudescence detection and control	• Guidance on surveillance and recrudescence<br>• Inter-country sharing of surveillance data/experiences<br>• Advocacy

A CHANGING CONTEXT FOR ONCHOCERCIASIS CONTROL

Since the establishment of APOC in the mid-1990s, a number of important changes have occurred in the contextual environment in which APOC presently operates. The health policy environment has changed significantly since the inception of APOC, including a major shift in the architecture of international aid, and hence the future of onchocerciasis control activities requires reassessment.

The new contextual environment in which APOC operates brings both threats and opportunities for the future of onchocerciasis control. These changes include, but are not limited to, the following:

- International commitment to the Millennium Development Goals (MDGs) where health features prominently in the targets to be achieved, as a measure of poverty reduction. Despite the emphasis on the "big three" (TB, HIV/AIDS, and malaria) combating other diseases is included in the MDG targets. The UK Commission for Africa in early 2005 specifically addressed the need to provide increased funding for the NTDs as a group, given the donated products available, the limited costs of the interventions, the efficacy of the drugs, and their broader impact.

- The African region has experienced an increase in the number of countries seen as fragile or failing states and directly or indirectly affected by conflict. Conflict has affected and still affects 3 out of 11 former OCP countries, and 7 out of 19 APOC countries are also severely hampered in their onchocerciasis control efforts because of the effects of recent or ongoing conflicts.

- The rapid spread and expansion of the HIV/AIDS pandemic, together with an increasing concentration on the "big three" in priority setting, both by MoHs and donors, and the establishment of specific funding mechanisms by the international community (the Global Fund, PEPFAR, and the President's Malaria Initiative), have led to a major emphasis on these diseases. The resulting re-deployment of human capacity to follow the funding flows, albeit in the short-term, may result in neglect of existing programmes and systems.

- An increased emphasis now exists on budget support and sector-wide approaches, with the accompanying trend to decentralize health systems, implement health sector reform, and develop basket funding for the health sector. These measures have been taken to strengthen country ownership and decentralized governance, increase donor coordination and reduce transaction costs. There is still considerable debate about the impact of these policies on critical measures of health for the poor.

- In parallel to these developments, several international initiatives have been created to open new channels for resourcing the social sectors. These include HIPC initiatives, the Medium Term Expenditure Framework (MTEF), and recent debt relief agreements allied to the creation of an International Finance Facility for childhood vaccines. These changes make the need for country-level advocacy for onchocerciasis control more important.

- The number of public-private partnerships to address health issues over the last decade has increased; however, a call for the harmonization and realignment of these partnerships was made in the Paris meeting of the High Level Forum. NTDs were addressed in this document as appropriate for such harmonization. Major donors will be looking towards proactive attempts to address these issues given their concern over transaction costs and the need to reduce the number of 'financial instruments' through which donor money is directed.

- "Competition" at national and local levels with other disease control programmes for resources has greatly increased leading to a scarcity of health services reaching the periphery. An urgent action to ensure sustainable improvements in health services and to promote synergy is necessary to prevent fragmentation of efforts and human resources.

- A major change since 1997 has been the donation of albendazole by GlaxoSmithKline and the extension of the Mectizan donation by Merck & Co., Inc., for the elimination of lymphatic filariasis (LF) in Africa, as well as the commitment of Pfizer Inc. to provide azythromycin (Zithromax®) for the elimination of trachoma as a public health problem globally.

These changes in context underscore that APOC now operates in a radically different environment than when the programme was conceived. These contextual changes present opportunities in terms of decentralization, basket funding, and health systems approaches, but also constrain prioritization of onchocerciasis control through competition for funds as well as the pressure on human resources in the health sector. These changes shape the strategic choices that face APOC and will determine the success of developing and sustainable national programmes for the future.

ISSUES FOR CONSIDERATION

During its discussions with members of the TCC, CSA, NGDOs, countries, other stakeholders and APOC management, the Group confirmed that the four core issues identified in the Terms of Reference were at the heart of decision-making regarding the future of onchocerciasis control in Africa.

Before discussing the four issues individually, the Group wishes to emphasize the successes of APOC and the important contributions it has made towards onchocerciasis control in Africa through the establishment of CDTI in countries throughout the APOC region. As a result, the Group recommends:

Recommendation 1: APOC's main objective of establishing sustainable national onchocerciasis programmes in all countries where needed should be maintained and endorsed.

**Issue 1: Extend APOC Programme Duration and Strengthen Capacity**

The first major issue is that of extending the duration of the APOC programme and strengthening its organizational capacity to respond to the changes in the context of onchocerciasis control. The Group recognizes the major achievements to date and would like to emphasize the progress APOC has made toward achieving its goals and mandate. In spite of this the Group recognizes that, for reasons largely beyond the control of APOC, one-third of the projects in APOC will not be sustainable by the original deadline of 2010. As of 2006, eight projects have not yet begun. If these programmes were to lose the support of APOC during the early phases of implementation they would be unlikely to reach the goal of sustainability.

A major obstacle to the ability of APOC to achieve its goal has been the impact of conflict on onchocerciasis control, which has occurred in a number of APOC countries. In regions of conflict, a fractured political climate has led to postponement, interruption, or the slowing of project implementation, resulting in a need for extra time and money in order to establish successes.

Loiasis has also been an important obstacle to more rapid implementation of national programmes in areas co-endemic with onchocerciasis because of Severe Adverse Effects (SAEs) that have followed treatment with ivermectin, in particular in individuals with high parasitic loads. This has had implications in terms of the time and resources needed to overcome the problem. Loiasis is a major reason for some countries (DRC and South Sudan) experiencing delays in programme implementation, although much progress has been made in the mapping of the disease through a simple rapid assessment technique (RAPLOA) elaborated by TDR. The prediction and management of SAEs is possible today owing to collaborative research between APOC, the Merck Donation Program, and Merck & Co., Inc., but further research and promotion of preventive and curative measures are still needed. A major need exists to train more staff and provide financial resources within the health care systems of loiasis co-endemic areas where onchocerciasis mass treatment is undertaken. An added complication in relation to

implementation, the loiasis problem will be overcome gradually, as multiple doses of ivermectin administered will eliminate high microfilarial loads due to loiasis. In areas where onchocerciasis mass treatment is not undertaken, loiasis will continue to constitute an obstacle to integrated chemotherapeutic interventions against lymphatic filariasis in Africa, which depends on the use of ivermectin plus albendazole.

The need for continued support was clearly stated in the external evaluation, which recommended an extension in APOC's duration to allow the programme to fulfill its objectives, finalize project implementation, and make a number of important shifts. These modifications include an increased emphasis on continued decentralization by APOC of programme responsibilities to country level and a shift from project to country support. While the integration of CDTI into health systems is primarily a country responsibility, APOC must act as a catalyst and provide necessary expertise. Decentralization has limitations during the initial phase of implementation when the APOC's financial and technical support is most intense and CDTI is being established.

Views on Issue 1

The Group considers that APOC can increase the effectiveness of its mandate if APOC's duration is extended and the time period is adapted to allow for more flexible responses on the basis of need. Countries progressing towards the sustainability goal, and where decentralization is advanced, will require limited input from APOC. Other countries because of conflict and SAEs associated with co-endemicity of onchocerciasis and loiasis will require additional resources and an expanded country-specific timeline.

Based on these observations, the Group feels that cessation of support to countries that have made a late start with CDTI would be detrimental and an unethical public health decision. The Group recommends that there is continuity of support to fulfill APOC's mandate.

APOC is a mature programme working as an established partnership and the Group believes it is appropriate to reconsider the management and governance structures of APOC. The solid partnership and governance structures were vital at the outset of the programme and continue to be important. The Group notes that the Committee of Sponsoring Agencies (CSA) is an effective executive body working on an inclusive and consensus-seeking basis reflecting the broad APOC partnership, including NGDOs and Merck & Co., Inc. This inclusive approach should be further strengthened.

The APOC Director has taken steps to delegate some of her tasks to senior programme management so that more of her time is available for key advocacy and leadership tasks. However, the changing demand for technical support and increased workloads suggests that staffing needs at APOC headquarters will need to be regularly reviewed and necessary adjustments made. The TCC has also adopted a more strategic approach to its meetings. The Group applauds this and considers it important to continue with this process. In the final stages of APOC, and in particular in preparation for a more flexible and sustainable model for onchocerciasis control, the Group thinks that a reduced number of statutory meetings and a more strategic approach will benefit the organization.

One strategic issue that APOC may need to address in the coming years is that of stopping ivermectin treatment after a prolonged period of CDTI. If the ongoing studies in Mali and Senegal (see page 5) provide evidence that onchocerciasis transmission can be eliminated with long-term ivermectin treatment, then APOC will need to provide technical guidance to countries on where and when treatment can safely stopped.

The Group recommends:

Recommendation 2: APOC operations should be extended to 2015 to enable it to fulfill its original objectives.

- APOC should complete the establishment of CDTI projects in all countries;
- APOC should develop an exit strategy which includes effective long-term support mechanisms for countries including mechanisms to provide the limited support to CDTI and onchocerciasis surveillance that will remain necessary after the programme has come to an end.

Recommendation 3: APOC should take on the additional objective of developing the evidence base to determine when and where ivermectin treatment can be stopped, and provide guidance to countries on how to prepare for and evaluate cessation of treatment.

- The JAF is asked to endorse this new objective recognizing that at present there is insufficient knowledge about when and how mass treatment can be stopped;
- APOC should develop a plan, including the excluding resource requirements, to monitor progress in selected areas where cessation of ivermectin treatment has occurred.

Issue 2: CDTI integration and co-implementation of additional health interventions

The term ”integration” is used in a number of different ways when discussing onchocerciasis control. To avoid confusion regarding this term, the Group uses the word “integration” when referring to the inclusion of CDTI activities in the broader health system with strong community involvement. The Group uses the word “co-implementation” when referring to the strategy of bringing two or more community distribution initiatives together to increase efficiency and avoid fragmentation. Integration of CDTI activities in the broader health system is important for sustainable onchocerciasis control and also for the sustainable co-implementation of interventions.

A number of major pharmaceutical companies (Merck, GSK, Johnson & Johnson, Pfizer, Sanofi-Aventis and Novartis) have donated drugs for neglected diseases. These commitments allow large populations of poor people to receive a broad spectrum of important and efficacious products at low cost. The cost of these interventions has been estimated at less than $1 USD per person, per year. The Commission for Africa recommended that donors should ensure that there is adequate funding for the treatment and prevention of parasitic diseases and micronutrient deficiency. Governments and global health partnerships should ensure that this is integrated into public health campaigns by 2006.

Recent evidence suggests that additional health benefits, such as other preventive chemotherapies, bed nets and immunizations, can also be delivered in a cost-effective manner

using a CDTI strategy. Given APOC's proven success in reaching the most under-served communities in Africa through CDTI, and the recognition that deployment of co-administered drugs and other health interventions can bring broad health benefits, the Group discussed at length how such opportunities could be implemented in the context of APOC and the future of onchocerciasis control in Africa.

The vision behind the CDTI strategy is that, wherever possible, CDDs work in close collaboration with the Primary Health Care services. APOC acknowledges that sustainability in onchocerciasis control is dependent upon government and NGDO commitment as well as on community ownership. The APOC definition of sustainability is that, "CDTI activities in an area are sustainable when they continue to function effectively for the foreseeable future, with high treatment coverage, integrated health care services, and strong community ownership, using resources mobilized by the community and the government." A critical requirement is to monitor the evolution and impact of CDTI as financial support to APOC is slowly phased out over time.

In areas where primary health infrastructure is weak and/or under-resourced, CDTI is working in many cases despite these challenges. For eco-geographical reasons onchocerciasis foci are among the areas worst served by formal health services. Hence, access of these populations to quality health care is minimal if not non-existent. In these situations CDDs provide access to one of the few health interventions available. From a public health perspective, the idea of coordinating or co-implementing the provision of two or more community directed interventions is particularly attractive in these types of settings. CDTI is a potential starting point for additional disease prevention and control activities where there is extensive co-endemicity and hence polyparasitism (multi infections in one individual).

In countries where primary health care infrastructure works reasonably well, the Group believes that many public health benefits will also accrue from integration and co-implementation. The Group contends that the policies encouraging and guiding integration and co-implementation will have benefits that extend beyond onchocerciasis control activities by providing increased accessibility to health care services at a minimal recurrent cost of significantly less than $1 USD per person, per year.

The Group acknowledges that much has already been realized, with coordination and co-implementation of multi-interventions occurring at the district and community levels in many former OCP and APOC countries. These activities are currently being driven by district or community-specific needs and contexts rather than national policies or global coordination mechanisms. Recent examples discussed by the TCC included treatment of lymphatic filariasis, vitamin A distribution, schistosomiasis control via chemotherapy, Guinea worm eradication, immunization programmes, trachoma treatment, malaria bed net distribution, and an updated census. Community-directed treatment strategies are the platform for these co-implementation efforts, suggesting a key role for APOC as a facilitator of the process. An outstanding advantage of APOC in the organization of CDTI programmes is its census of rural communities, unique in its completeness due to regular annual updating.

Recent studies suggest that co-implementation with other treatment distribution programmes can have important benefits for onchocerciasis control, including increased coverage and cost savings. Other benefits not specific to onchocerciasis control may also be realized. In particular, various types of activities in the earliest stages of implementation may benefit from existing networks for CDTI. Similarly, onchocerciasis control may benefit from long-term commitments made by these other programmes.

Figure 2: Co-implementation at the National Level - The Case of Mali

In Mali, CDTI is implemented under the supervision of the Chief Nurse of the Community Health Centre, who in turn works under the supervision of the District Health Team. Ivermectin distribution is a community directed initiative fully integrated into the health system. Other health interventions including bednet distribution, mass drug administration of albendazole for lymphatic filariasis (in combination with ivermectin), azythromycin for trachoma, and praziquantel for schistosomiasis use the same procedure with a high level of community involvement backed up with supervision by staff from the Community and the District Health Centres. Funding for these activities is principally provided by the International Trachoma Initiative, the Schistosomiasis Control Initiative, NGDO partners, and the government of Mali.

Views on Issue 2

The Group believes APOC has important expertise to be shared in relation to community directed distribution and public-private partnerships. The group recommends that APOC should advocate and disseminate its experience in CDTI and pursue an expanded strategy on co-implementation with the programmes that have thus far been shown to be compatible with APOC's mandate. Expansion activities should go hand in hand with additional applied research and experimentation with different forms of co-implementation, as many questions remain unanswered. APOC, with its substantial experience with CDTI, could be the focus of such an operational research agenda.

The Group also realizes that in addition to the many advantages and efficiencies that can potentially be reached through well coordinated co-implementation there are also a number of potential risks in pursuing this strategy and that the issues are seldom simple. One concern is that co-implementation with disease control programmes that are less well funded may have an unfavourable effect on funding for onchocerciasis control. Another concern is competition among CDDs arising between CDTI and better-financed programmes with the potential effect of losing sight of specific APOC objectives and operational requirements.

Table 3: A SWOT analysis of APOC's potential role in co-implementation

**STRENGTHS**	**WEAKNESSES**
• Evidence base on community based disease control<br>• Track record and experience with CDTI<br>• Community involvement<br>• Partnership experience<br>• Synergistic effects of treatment<br>• Long-term commitment from pharmaceutical partners including the MDP<br>• Inter-country organization<br>• Ongoing support for operational research<br>• Linked to NGDOs/TDR	• Current programme approaching end<br>• Overload of staff<br>• Under-funding of other possible partner programmes<br>• Limited human resources (APOC and country-level)<br>• Limitations of project procedure and approach<br>• Onchocerciasis and NTDs in general are not high enough of a priority in country health plans
**OPPORTUNITIES (BENEFITS)**	**THREATS (RISKS)**
• Increased awareness of neglected tropical diseases<br>• Potential public health impact on large populations (up-scaling)<br>• Impact on poor populations<br>• Quick win/MDGs<br>• Cost effective<br>• Access to the community<br>• AFRO support and interest	• Diluted priorities<br>• Financial insecurity of future partners<br>• Incentive issues<br>• Conflicting policies at global and national levels<br>• Overload on programme capacity<br>• Potential for loss of refined monitoring<br>• Reduction in the quality of data<br>• Loss of specificity<br>• Over-emphasis on HIV/AIDS-Malaria-TB

The Group reflected in depth on the options relating to co-implementation considering APOC's strengths and the added value it can bring to other disease control programmes and opportunities presented but also considering its specific strengths and geographical foci. The Group has also reflected on what can and should be expected from a regional/global programme and what falls properly within the mandate of national ministries of health and national programmes. The Group has identified a number of domains in which it feels that APOC can make an important contribution to integration and co-implementation.

Table 4: Potential Role of APOC in Co-Implementation

Domain\Level	International/Regional	National
**Policy**	• Advocacy for national policy on integration and co-implementation of community based intervention programmes using CDTI to promote MDGs<br>• Promote use of evidence for policy development<br>• Advocacy for financing for integration through alternative financial instruments<br>• Advocacy directed at multilateral/regional organizations	• Facilitating role for policy development<br>• Advocating for financing for integration and co-implementation through alternative financial arrangements
**Organizational**	• Coordination with:<br>o Health intervention programmes and NTD partnerships<br>o NGDOs<br>o Research organizations	• Coordination (through NOTFs) with:<br>o Health intervention programmes and partnerships<br>o Private sector<br>o Research Organizations
**Activity**	• Research and evaluation to generate evidence base for integration and co-implementation<br>• Disseminate evidence for integration and co-implementation	• Financial and technical support for:<br>o Implementation of integrated CDTI<br>o Mapping of co-endemicity<br>o Monitoring and Evaluation<br>• Contributing to the development of human resources for integration and co-implementation<br>• Encourage operational research on integration and co-implementation and dissemination of lessons learnt

The above matrix[8] is designed as a template for proposing potential future roles of APOC in assisting countries to develop policies for integration and define practicalities for co-implementation in their own settings. These activities are consistent with the mandate outlined in APOC's current memorandum. The Group thinks that at the international and national levels, APOC and other NTD partnerships can work together towards the objectives of harmonization of partnerships and programmes.

The Group recommends:

Recommendation 4: APOC should promote integration and co-implementation of interventions with CDTI to effectively provide multiple health benefits to large populations.

[8] The above matrix is based on a framework developed by Grépin and Reich.

- APOC should advocate for integration of CDTI into strengthened health systems;
- APOC should develop plans for facilitation, advocacy, and exchange of best practices;
- The CSA and the APOC Partnership should encourage joint financing and advocacy for Neglected Tropical Disease (NTD) partnerships;
- APOC, TDR, and partners should continue and strengthen operational research on co-implementation;
- The CSA should invite other NTD partnership representatives to JAF to promote harmonization of partnerships.

ISSUE 3: APOC's mandate should be broadened to include all onchocerciasis endemic countries in Africa

OCP ended in 2002, 28 years after its inception. OCP's success in controlling onchocerciasis in West Africa has been remarkable. At the start of the programme an estimated 2.5 million of the programme area's 30 million inhabitants were infected and 100,000 blind. Today it is estimated that 600,000 cases of blindness in the region have been prevented, and an estimated 25 million hectares of arable land regained. The programme was highly cost effective with an economic rate of return of 15-20%, costing less than $1 per annum per protected person.[9]

Most former OCP countries have shown commitment to maintaining the gains of OCP since closure in 2002. CDTI has continued where indicated with excellent coverage in Burkina Faso, Mali, Niger, and Senegal resulting in satisfactory epidemiological parameters. Enhanced CDTI has also been implemented satisfactorily in the SIZ areas of Benin, Guinea, and Togo. The epidemiological situation has improved in most of the areas and Guinea promptly identified and intervened when recrudescence was detected in border villages with Sierra Leone, where CDTI reactivation after the conflict situations has shown initial promise. Non-SIZ and forest onchocerciasis extension areas are also being treated in these countries in line with the epidemiological situation. Co-implementation of onchocerciasis and lymphatic filariasis control with mass drug administration has commenced or is being planned where co-endemicity exists in most countries. Onchocerciasis surveillance to prevent recrudescence is continuing, and has been integrated in national disease surveillance systems in most countries, though challenges (mainly budgetary) remain.

Despite these achievements, maintenance of the OCP gains is sensitive to changes in the political environment, to shifting health priorities, and in particular, to the conflicts which have affected and continue to affect some of the former OCP countries.

The Group identified three specific concerns that have become pressing since the closure of OCP:

- The situation in fragile states and former OCP countries affected by conflict;

[9] Kim A & Benton B (1995) Cost-Benefit Analysis of the Onchocerciasis Control Program (OCP). The World Bank, Washington, DC.

- The situation regarding OCP Special Intervention Zones;
- The need for some continued liaison and support for onchocerciasis control activities in former OCP countries.

The Situation in Fragile States and Former OCP Countries Affected by Conflict
A number of former OCP countries face major challenges as a result of conflict and political instability. In these countries, civil disturbance and consequent migration have caused major disruptions in onchocerciasis control operations, and many of the structures and systems established by OCP have disappeared. The conflict situation has prevented continuation of control activities in Sierra Leone, Côte d'Ivoire, and Guinea Bissau.

The epidemiological situation in Sierra Leone is very alarming, as some indicators have reverted to the pre-control situation of a decade ago. CDTI alone (without any vector control operations) has been established countrywide with the support of the OCP Trust Fund and NGDOs. The results of these renewed efforts are not yet known. The SIZ evaluation suggested that control efforts would need to start from the beginning stages in certain post-conflict areas. In Côte d'Ivoire the epidemiological situation in the north of the country is unknown and as a result the potential threat to the core of the former OCP cannot be assessed. Burkina Faso and Mali are those countries most likely to be affected by any recrudescence in Côte d'Ivoire.

The Situation Regarding OCP Special Intervention Zones
OCP ended in December 2002, having achieved the objectives of eliminating onchocerciasis as a disease of public health importance and as an obstacle to socio-economic development throughout practically all of the programme area. However, in limited and defined river basins, the entomological and epidemiological situation remained unsatisfactory after several years of combined vector control and ivermectin treatment. These areas therefore qualified for "Special Intervention" and were referred to as the "Special Intervention Zones (SIZ)." The main strategy of control was CDTI applied until 2012, reinforced to achieve maximum geographic and therapeutic coverage. CDTI was to be complemented with vector control for 5 years in Upper Oti and Upper Oueme river basins in Togo and Benin. Epidemiological and entomological surveillance were key tasks. The SIZ activities were to be carried out using undisbursed residual funds held in the OCP Trust Fund. The objective of creating the SIZ was to achieve a reduction in onchocerciasis epidemiological parameters (prevalence and community microfilarial load) to a level that would satisfy the OCP standards. Considerable progress has been made in these areas, but the mid-term review of SIZ suggests that the SIZ will continue to need a degree of support, especially in relation to surveillance and the implementation of planned CDTI activities, to achieve maximum coverage. After 2007, national onchocerciasis control programmes in the SIZ areas will require the same type of external support that has proved necessary in non-SIZ areas of former OCP countries. The case of Sierra Leone, which is currently re-starting CDTI activities, is of particular importance.

Cross Border Issues
Cross-border issues relating to transmission will remain problematic but APOC initiatives for cross-border onchocerciasis control coordination have already been created in the former OCP area often at the behest of APOC/SIZ management. Regular meetings have been held between Guinea/Sierra Leone/Liberia, Togo/Bénin, and Bénin/Nigeria.

In this context APOC has demonstrated leadership by coordinating sub-regional bodies and inviting former OCP national onchocerciasis control programme coordinators to attend annual SIZ meetings. Cross-border initiatives as well as separate meetings for national coordination bodies need to be continued on a regular basis but require external support.

The Need for Continued Support for Onchocerciasis Control Activities in former OCP Countries

In general the decentralization of activities and responsibilities to the national level in former OCP countries has been successful. A number of countries have demonstrated that programme sustainability can be achieved by integration into national health systems and that funding from both government and external sources can be maintained. However, in some cases funding from NGDOs has been uneven and unpredictable, and in most countries maintaining regular government contributions poses a challenge. The following issues are important considerations:

- Even those countries that are maintaining onchocerciasis control are concerned about longer-term success in the absence of a forum for exchange or for advocacy to maintain political motivation and resource allocation for this issue.
- Due to the rapid turnover of personnel and pressure on human resources in the sector there are difficulties in maintaining a core of onchocerciasis expertise. This is a formidable challenge in countries severely affected by onchocerciasis, which are often the most underprivileged and ill-resourced.
- Closure of OCP has in some cases resulted in policy makers concluding that "the problem is solved." Given the pressure on health budgets this thinking provides a rationale for reducing commitments and giving less priority to the disease. This underscores the need for advocacy and for a process of monitoring to ensure countries continue to meet their commitments.
- The gains of OCP could be lost without a relatively small investment for surveillance and providing technical support. Failure to maintain trypanosomiasis control resulted in epidemics in Central Africa as health services disintegrated, resources declined, and mobile teams were abandoned. The resurgence of trypanosomiasis resulted in hundreds of thousands of deaths between 1970 and 2000. Maintenance of surveillance is as much a prerequisite for onchocerciasis as it was for trypanosomiasis. Failure to invest in this maintenance phase activity would mean that health policy makers have ignored the lessons of earlier programmes.
- The Multi-Disease Surveillance Center (MDSC) was originally envisaged as playing an important role in supporting surveillance for former OCP countries. Lack of resources unfortunately limits the extent to which the MDSC can take on this role, but it is expected that with a new strategy from WHO/AFRO this situation will be improved. Progress on this needs critical monitoring, as support to surveillance is essential for control.

Figure 3: Risk of Disease Recrudescence in former OCP Countries - The Case of Côte d'Ivoire and Ghana

Côte d'Ivoire: is a non-SIZ country which has been in conflict since 2002. The onchocerciasis control programme has lost all of its equipment and some trained personnel. CDTI activities have been interrupted. There has been no information available from the northern part of the country since the onset of the conflict. Epidemiological surveillance carried out in 2005 in the southern extension showed high prevalence rates of up to 41%. The unsatisfactory epidemiological situation could deteriorate further unless peace returns and control activities are re-established, posing a threat of recrudescence to Guinea, Mali, and Burkina Faso, where effective control exists.

Ghana: is a politically stable country with SIZ and non-SIZ areas, where co-implementation of LF and onchocerciasis control with ivermectin is being implemented in co-endemic districts. Geographic and therapeutic coverage rates in the SIZ areas have been inadequate since 2003. In 2005, only 3 out of 7 districts carried out ivermectin distribution. Results of epidemiological surveys suggest an increasing trend of disease prevalence over the past three years. Entomological surveillance carried out in 2005 showed *O. volvulus* infectivity rates of 1.82/1000 flies in Asubende in the Pru basin. Although this is less than 5% of the pretreatment level of infectivity, it is 4 times the acceptable threshold. The situation should be addressed urgently. Ghana also represents a threat to neighboring countries in terms of recrudescence.

Views on Issue 3

The Group sees no reason to maintain the distinction between former OCP and APOC countries in thinking about the future of onchocerciasis control since ivermectin distribution based on CDTI is a tool available to all countries and underscores the need for onchocerciasis control strategies in all countries where needed. Policy decisions historically resulted in the development of the two regional programmes known as APOC and OCP, but in the current situation there is no reason to maintain the distinction between two "onchocerciasis regions". The vector is unaware of national boundaries and people migrate regularly in border regions. The risk of recrudescence is real in the absence of satisfactory surveillance. The current APOC Memorandum leaves room for the JAF to invite other countries to join APOC. The group has received legal advice indicating that former OCP countries can be included in APOC through the invitation of the JAF and the agreement of each country. The recommended expanded mandate must take into account the ability to intervene and support renewed activity where and when it is necessary on the basis of epidemiological imperatives.

The Group is of the opinion that advocacy and flexible technical support would do much to protect the gains of OCP and of APOC. The Group feels that a number of partners could play a role in this endeavor including MDSC, WHO AFRO and APOC itself. The Group also considers that a clear plan should identify the roles and responsibilities of these actors and that funding should be secured (where necessary) to ensure that each of these players can fulfill its role. The Group believes that much could be achieved with a relatively low level of investment.

The Group Recommends:

Recommendation 5: APOC's mandate should be extended to include all onchocerciasis endemic countries in Africa where the epidemiological situation requires sustainable CDTI.

- The JAF is recommended to endorse this extended mandate which can be executed within the terms of the existing Memorandum;
- APOC will thus ensure continuity of support to Special Intervention Zones (SIZ) and former OCP countries where sustainable CDTI still needs to be established;
- APOC partners should, promote actions to strengthen the capacity of the MDSC to fulfill its anticipated role in relation to onchocerciasis surveillance.

Issue 4: Strategic options to address the future financial needs of the programme

The Group discussed a number of issues in relation to the financing of the future needs of onchocerciasis control. The discussion focused on the future funding of sustainable country programmes and the funding for the core functions performed by APOC in support of country programmes. The Group anticipated that a greater proportion of financial resources for onchocerciasis control will be provided by the countries but considered that the maintenance of core Trust Fund financing was required given the continuing strategic regional role that APOC will continue to play.

The financing of APOC has followed the mechanism of OCP. The donor funds are deposited in an APOC-specific World Bank Trust Fund. Contributions are not earmarked and are for the sole use of APOC activities as outlined in the Memorandum of Understanding. This financing model has proved to be a robust and fully accountable mechanism with funds managed by the World Bank and disbursed to APOC as required. Budgets are approved annually by JAF and at no stage has the External Auditor recorded any problem in the use or management of the Trust Fund. The present Memorandum allows use of Trust Fund monies to be used for other disease control activities within the framework of CDTI so there is a high degree of flexibility for APOC should donors wish to use the APOC Trust Fund as a financial instrument to support wider neglected disease control activities.

Views on Issue 4

The Group believes that APOC should build on the support of traditional donors and use the proven existing mechanism of the Trust Fund as an instrument. At the same time the Group thinks it essential that APOC diversify its sources of funding and identify and use new opportunities, particularly those resulting from increased recognition of the importance of and possibility of controlling NTDs. The group thinks that integrated disease control initiatives and global funds will be important additional sources of funding or co-financing. These financing sources will probably not be channeled through the APOC Trust Fund but are more likely to be routed directly to countries to support integrated NTD control programmes. APOC should be encouraged to explore these opportunities with country programmes.

The long-term sustainability of onchocerciasis control will depend to a large extent on the commitment of national and local governments to allocate from the national budgets the necessary funds for onchocerciasis control as a priority in national and district health plans. In spite of the limited resources available the Group thinks that securing country commitment is vital.

APOC (and OCP) have been successful in advocating for the need for countries to take on the responsibility for CDTI and it is important that this advocacy continues. There is a strong case for addressing Ministries of Finance as well as Ministries of Health in these advocacy efforts given their commitments to the MDGs and the recognition of the need to control poverty promoting conditions.

A number of additional financial instruments have been developed to support the social sectors (e.g. HIPC). These instruments help to increase the fiscal space and make it easier for countries to finance the provision of a number of inexpensive cost-effective interventions such as the control of onchocerciasis and other neglected tropical diseases.

The Group recognized the significant contributions made by NGDOs not just as partners in implementation but also in terms of the contributions made to financing APOC activities at the country level, managing projects, and contributing to the NOTF in technical assistance. The Group recognized that some NGDOs have had difficulty in continuing their financial commitment to APOC but encouraged them to maintain existing levels of support critical to sustainability of country programmes and hoped that it would be possible to involve new NGDOs in support for CDTI.

The Group also recognized the substantive contribution of Merck & Co. Inc., in providing Mectizan and in support of the Mectizan Expert Committee as an independent advisory body.

The Group Recommends:

Recommendation 6: Financial planning and fundraising for onchocerciasis control should build on existing mechanisms and traditional donors but should also explore new funding opportunities particularly those offered in the context of NTDs.

- Financial planning and fundraising will need to take into account the need to secure country commitments to stable funding for sustainable country programmes;
- Financial planning and fundraising should cover the essential activities of the extension to 2015 to allow APOC to fulfill its core mandate and the additional activities identified in the strategic overview;
- Planning for an exit strategy should include costing of core regional functions that will be necessary post-APOC together with an overview of which partners and actors may be able to take on these functions and the necessary resource requirements.

CONCLUSION

The Group has examined and analyzed the many facets of onchocerciasis control based on a significant body of documentation including the most current scientific information and the overall public health and development landscape. It sees APOC as the prime institutional driver to reduce the public health problem of onchocerciasis, which is an avoidable impediment to the lives of millions of the poorest in Africa. Onchocerciasis control exists in a continuum of changing scientific evidence, national and global policies, politics, and financing priorities.

Global public health successes need recognition, celebration and investment; APOC deserves those accolades. It is a programme that has provided a paradigm shift in thinking through its development of CDTI; in the context of the cost-effectiveness of the product delivered; the numbers of poor people reached; the high coverage of previously un-served communities and the overall public health and health system benefits. These broad achievements deserve greater recognition and support by the international health community. APOC is one of a few successful vibrant and effective partnerships on the public health agenda.

The Group concluded that the future of onchocerciasis control in Africa was entering a critical phase and that it was necessary to make clear and strategic provisions to allow APOC to fulfill its mandate and to secure the gains of OCP and APOC for the future.

Financial constraints, political instability and complications with SAEs have led to delays in the implementation of CTDI in a number of countries. The Group is of the view that an extension to 2015 is necessary to successfully establish CDTI in all countries and also to develop the evidence base around if and when ivermectin treatment can be stopped.

The Group is of the view that APOC should have a wider strategic role in promoting co-implementation, and the lessons learned of co-implementation with other disease control initiatives through its flagship CDTI approach.

The current Memorandum is one that allows for flexibility of operations. The Group has been informed that the extension of the geographical coverage of APOC and the inclusion of other countries in need of support does not require the development of a new memorandum. The Group believes that this flexibility should be used to enable APOC to take on a more flexible approach to onchocerciasis control in Africa.

The Group believes that while financial planning and fundraising for onchocerciasis control should build on existing mechanisms and traditional donors, new funding mechanisms, such as those that have appeared in recent NTD initiatives, should be actively pursued.

The Group is convinced that the future of onchocerciasis control depends on continued community involvement, concerted national commitment and coordinated Pan-African support. The Group does not argue for an indefinite commitment to APOC but strongly believes that APOC's mandate should be extended and broadened until 2015 to allow it to respond flexibly to the needs of all countries in which mass treatment is needed to establish sustainable CDTI programmes to protect the investments already made, to encourage prioritization of

onchocerciasis and other neglected diseases through sustainable country financing and ensure the maintenance of effective surveillance systems. This necessitates the development of an exit strategy involving a clear definition of roles and responsibilities and securing commitments to inter-country support mechanisms after APOC. The plan for transition should be in place before APOC ends.

WORKING GROUP MEMBERS

- Prof. Adenike Abiose, Sightcare International, Ibadan, Oyo State, Nigeria,
- Mrs. Catherine Hodgkin, Royal Tropical Institute (KIT) Netherlands (Chairperson)
- Prof. David Molyneux, Lymphatic Filariasis Support Centre, Liverpool School of Tropical Medicine, Liverpool, UK
- Dr. Bernard Philippon, Medical Entomologist, Department of Society and Health, Paris, France
- Prof. Michael Reich, Harvard University
- Dr. Jan H.F. Remme, Special Programme for Research and Training in Tropical Diseases, WHO Switzerland
- Dr. Bjorn Thylefors, Mectizan® Donation Program
- Dr. Mamadou Souncalo Traoré, Directeur National de la Santé, Ministère de la Santé, Bamako, Mali, (Vice Chairperson)

- Mrs. Karen Grépin, Harvard University (Rapporteur)
- Ms Elissa Klinger, Harvard University (Rapporteur)

ANNEX 1: TERMS OF REFERENCE

Working Group on the future of onchocerciasis control in Africa

----

Terms of Reference

1. Introduction and Background

1.1 The objective of the African Programme for Onchocerciasis Control (APOC) is to establish by 2010 effective and sustainable Community-Directed Treatment with Ivermectin (CDTI) systems to protect 102 million people at risk in 19 countries which are outside the 11 countries of the former Onchocerciasis Control Programme in West Africa (OCP). These CDTI projects are being established to become entirely self-sustaining within the 1996-2010 time frame. The Programme also aims at eliminating the vectors transmitting the parasite in selected circumscribed foci. The attainment of this objective is expected to lead to the elimination of Onchocerciasis as a disease of public health and socio-economic importance throughout Africa.

1.2 The principal tool for controlling and eventually eliminating onchocerciasis as a public health problem throughout the Programme area is ivermectin (Mectizan ®) provided free of charge by the manufacturer, Merck & Co., Inc. During Phase I (1996-2001) of APOC operations, the Programme approved 59 projects, which had close to 25 million persons under ivermectin treatment at the end of 2001. By the end of Phase II (2002-2007) and the Phasing-out Period (2008-2010), 52 projects will have been approved, bringing the total number of CDTI projects approved to 111. After 2010, approximately 90 million people will be treated on a yearly basis to protect an at-risk population of 102 million persons.

The community-directed treatment with ivermectin, the control strategy of APOC, is backed by an effective partnership and adequate resource mobilization. Empowerment of communities and their involvement in the delivery of Ivermectin is a key feature of the Programme.

APOC is a regional Programme with its headquarters based in Ouagadougou (Burkina Faso) providing for policy harmonization, and promoting collaboration and coordination among the Participating Countries. APOC partnership is governed by a Memorandum of Understanding (MoU), signed by all partners in December 2001. The MoU sets forth the financial arrangements and the institutional and operational aspects of the Programme. The MoU for Phase II and the Phasing-out period authorizes the Programme to approve funding for projects combining community-directed treatment with ivermectin with other community-based programmes within the geographic scope of the Program.

1.3 The cost of APOC operations during Phase I amounted to US$56 million from donor contributions, with an additional 25% provided by the Non-Governmental Development Organizations (NGDOs) and the African Participating Countries. The corresponding donor cost of Phase II and the Phasing-out Period is estimated at US$79.2 million. Funding for Phase II

(US$68 million) has been secured. The present financial gap of the Programme for the Phasing-out period is around US$9 million.

1.4 The findings of the External evaluation of APOC, conducted in 2005, highlighted the contribution of the Programme to the improvement of national health care delivery systems of Participating countries through increased access to health services for communities "beyond the end of the road"; involvement of communities in health care provision; and increased capacity of MOHs, communities and local NGOs. Another finding of the External evaluation is that "APOC is steadily moving towards its objectives, but still facing substantial challenges to continued satisfactory completion. Most of the issues identified are not new, and there is considerable ongoing and planned work aimed at finding solutions to these issues, such as (Serious Adverse Events) SAEs". On the elimination of the vector by using environmentally safe methods, the evaluation concluded, "the goal has been attained in one focus and is achievable in another." However there are delays, particularly in conflict or post-conflict countries where a number of projects have not even started. In view of the above, the evaluation team recommended the extension of APOC beyond 2010 as the "Programme will not be able to achieve its objective of establishing effective and self-sustainable CDTI throughout the endemic areas within the geographic scope of the programme, by the year 2010."

1.5 Furthermore, the team strongly felt that "the key role and contribution of APOC towards the elimination of onchocerciasis as a disease of public health and socio-economic importance throughout Africa and so contribute to improving the welfare of its people should be continued and enhanced". At the Regional/Global level, APOC will be needed to coordinate efforts in such areas as research (the effect of CDTI on interruption of the parasite, SAEs, resistance to ivermectin, and new experiences in organizing CDTI), project evaluation, impact assessment, advocacy, and continued exchange of information between countries.

1.6 The mid-term evaluation of the Special Intervention Zones of the ex-OCP countries, which was also conducted in 2005, indicated the need to ensure that the achievements of OCP are sustained. The evaluation team called upon countries concerned, WHO and the World Bank to pay particular attention to the situation in Sierra Leone, the critical role of surveillance in ensuring sustainability of the achievements in ex-OCP countries, and the urgent need for an inter-country mechanism that would liaise closely with APOC in overseeing onchocerciasis control activities in ex-OCP countries and throughout Africa.

1.7 At the same time, considering the success of the pilot projects in the use of CDTI as a vehicle for other health interventions, there is a growing interest at the national and international levels to adopt the community-directed approach of APOC for integrated delivery of compatible health interventions, and intensified control of some of the "neglected" tropical diseases.

1.8 At the 11th session of the Joint Action Forum (JAF), based on the current challenges and constraints of the Onchocerciasis Control Programs (OCP and APOC), and taking into consideration the results of the external evaluation of APOC and the Mid term evaluation of the Special Intervention Zones (SIZ) of the former OCP, the Committee of Sponsoring Agencies (CSA) made a presentation to the Forum on a vision for the future of APOC supported by four strategic actions:

- Extension of APOC to enable all projects to be brought to a satisfactory conclusion. The year 2015 was proposed as the new target, considering the time needed for all projects to receive adequate support from APOC Trust Fund, and also because this is also the target for the Millennium Development Goals (MDGs).
- Strengthening the performance of the Programme at all levels to enhance the likelihood that APOC will achieve its objective and target.
- Broadening the mandate of APOC to include ex-OCP countries to ensure that the achievements of OCP are maintained and sustained.
- Integrating onchocerciasis control with other compatible health interventions, especially the "neglected" tropical diseases, with the dual objectives to enhance the sustainability of CDTI and contribute to the achievements of the MDGs in APOC countries.

1.9 CSA also expressed the view that, although the burden of managerial and operational responsibility for implementation of integrated interventions falls on countries, APOC can play a significant catalytic role to accelerate the integration of compatible neglected diseases.

1.10 The Forum requested CSA to develop a position paper to be presented to a meeting of the partners to be convened in 2006 in an African country.

1.11 An independent advisory Working Group ("the Group") is, therefore, being set up to develop the paper requested by JAF, based on the Terms of Reference below.

2. Terms of reference

As recommended by JAF11, the Group is requested to reflect on the following points:

a) The future of the Programme until 2010 including the modalities of an eventual extension;
b) Modalities of continuing control activities in the countries (transfer, integration, funding and advocacy);
c) Geographical extension: APOC, SIZ areas (after 2007), ex-OCP countries and countries in conflict situation;
d) Eventual financing before and after 2010.

Specific issues to be considered by the Group are described below.

2.1 *Broadening the mandate of APOC to include ex-OCP countries*

2.1.1 To review the current mandate of the African Programme for Onchocerciasis Control and other onchocerciasis control activities being conducted outside the geographical scope of APOC; assess the effectiveness of the structures, technical, administrative and financial arrangements in place for the control of the disease throughout the African region.

2.1.2 To discuss the consolidation of APOC and ex-OCP into one programme.

2.1.3 To make recommendations for the priority areas that need to be focused on, with a view to maintaining and sustaining the achievements of oncho control in the ex-OCP countries before and after 2007.

2.2 *CDTI and integration*

2.2.1 To review the performance of health intervention projects that use CDTI as a vehicle as well as the challenges and lessons learned.

2.2.2. To review the integration policies and strategies of participating countries as well as the results of studies being conducted on the possibility of integrating CDTI with other health interventions to identify key factors that could enhance the process.

2.2.3. To assess the potential value added and the feasibility (weighing benefits against risks) of using the APOC structure as a catalyst for expanding integrated delivery of appropriate health interventions, especially neglected tropical diseases in onchocerciasis endemic countries.

2.2.4. To discuss the modalities of continuing the implementation of integrated projects, using CDTI as a vehicle in the countries (transfer, integration, funding and advocacy) and recommend interventions, which are most compatible and have the highest cost-benefit ratio.

2.2.5. To make recommendations on the future role of APOC on integration.

2.3 *Extending APOC Programme duration and strengthening its implementation capacity*

2.3.1. To review and assess progress made as well as the difficulties encountered in launching and implementing the 111 planned CDTI projects, based on the need for a minimum five-year financing from the APOC Trust Fund.

2.3.2. To assess the time needed to support all CDTI projects (particularly those in conflict and post-conflict areas) in order to achieve the objective and targets of the Programme..

2.3.3. To suggest, based on the mandate, objective and targets, current progress of the Programme and the above assessment, a realistic timeframe for reaching the objective of APOC as defined in para 1.1.

2.3.4. Make recommendations on the key measures (policy, organization, management, advocacy, funding, etc) to be implemented by APOC partners, APOC Management and by the various levels of the national health systems of Participating countries, to enhance the effectiveness of APOC operations in order to achieve this objective within the agreed timeframe.

3. Financing of the Programme

3.1. To review current availabilities of funding for APOC projects and assess the financial needs based on broadening the mandate and extending the duration of the Programme.

3.2. To suggest strategies for addressing future financial needs, if the mandate of the Programme is extended or broadened, including strategies for increasing domestic resource allocation from Participating countries.

4. Organizational aspects

4.1. The Group will consist of seven independent experts. CSA will finalize the composition of the Group, while ensuring that the required expertise is represented in the group, and arrange for secretarial support.

4.2. The working sessions of the Group shall start by March 13, 2006 and may include:

- A brainstorming session to be attended by the members of the Group, representatives of APOC partners, APOC management and TCC members, Chair and Vice Chair of JAF, representatives of selected countries (Nigeria and Cameroon in APOC area, and Burkina Faso and Sierra Leone in ex-OCP areas) in Ouagadougou- Duration 2 days.

- Two private meetings of the Group, including one in Ouagadougou just after the brainstorming session, and another before the CSA session scheduled in July 2006 in Paris.

- Presentation of the final draft report to CSA during its 114Th session in July 2006 (duration: 1 day) and presentation of the final report by two representatives of the Group to the partners meeting (duration: 3 days).

4.3. The materials to be used by the Group will be provided by APOC Management, APOC partners (WHO, World Bank, the Mectizan® Donation Program (MDP), the NGDO Group, bilateral donors and countries), and Members of the Technical Consultative Committee of APOC.

4.4. The activities of the Group will be financed from APOC Trust Fund. The total duration of the assignment is expected not to exceed 15 working days. However, the final nature and length of the assignment, and the practical arrangements will be set up after consultation between the members of the Group and CSA represented by APOC Director.

ANNEX 2: LIST OF CONSULTATIVE MEETING PARTICIPANTS

MEMBERS OF THE WORKING GROUP

1. Mrs Catherine Hodgkin, Royal Tropical Institute (KIT), P.O. Box 95001, 1090 HA Amsterdam, The Netherlands.
2. Dr Mamadou Souncalo Traoré, Directeur National de la Santé, Ministère de la Santé, Bamako, Mali.
3. Mrs Hilda Ausi Gondwe, Permanent Secretary, Ministry of Health and Social Welfare, Samora Avenue, P.O. Box 9083, Dar-es-Salaam, Tanzania.
4. Dr Frédéric Goyet, Chef du Bureau de la Santé, Président du Forum, Ministère des Affaires étrangères, 20, rue Monsieur, 75007 Paris 07 SP, France.
5. Dr Bjorn Thylefors, Director, Mectizan Donation Program, 750 Commerce Drive, Decatur, 30030 GA, United States of America.
6. Dr Adrian Dennis Hopkins, Medical Advisor, Regional Office, P. O. Box 58004-00200, Nairobi, Kenya
7. Prof Ekanem Ikpi Braide, Vice Chancellor, Chair of TCC, Cross River University of Technology, Office of the Vice Chancellor, CRUTECH, P.M.B. 1123, Calabar, Cross River State, Nigeria.
8. Prof. Adenike Abiose, Sightcare International, P.O. Box 29771, Secretariat Main Office Ibadan, Oyo State, Nigeria.
9. Mrs Karen Grépin, 21 Central Street, Auburndale, MA 02466, USA.
10. Dr Dominique Kyelem, Coordonnateur du Programme de lutte contre la lymphatique Filariose, Chef Service des Maladies Transmissibles, Ministère de la Santé, Ouagadougou, Burkina Faso.

INVITED NATIONAL COORDINATORS

11. Dr. Jonathan Yisa Jiya, Director, Health Planning, Research and Statistics, Federal Ministry of Health, Federal Secretariat, Phase III Ahmadis Bello Way, Abuja, Nigeria.
12. Dr Kupa Marcel Mukengeshayi, Coordonnateur National, Programme National de lutte contre l'Onchocercose, Avenue de la Justice No 36, Commune de la Gombe, RDC.

WHO/GENEVA

13. Dr Tony Ukety, NGDO Group Responsible Officer (GRO), Prevention of Blindness and Deafness, World Health Organization, 20 Avenue Appia, 1211 Geneva 27, Switzerland.
14. Dr Annette Kuesel, Scientist, OMS/TDR, WHO, 20 Avenue Appia, 1211 Geneva 27, Switzerland.
15. Mr Abdulai Daribi, AFRO/APOC Liaison Office, World Health Organization, 20, Avenue Appia, CH-1211 Geneva 27, Switzerland.
16. Dr Jan H.F. Remme, Coordinator, Science Strategy and Knowledge, Special Programme for Research and Training in Tropical Diseases, WHO/Geneva, 20 Avenue Appia, CH-1211 Geneva 27, Switzerland.

WORLD BANK

17. Dr Ousmane BANGOURA, Onchocerciasis Unit Coordinator, The World Bank,1818 H Street, NW, Washington D.C 20043

WHO/SIZ

18. Dr L.K.B. Akpoboua, DTL/SIZ, P.O. Box 549, Ouagadougou, Burkina Faso.

WHO/MDSC

19. Dr Yiriba Bissan, Entomologist, P.O. Box 549, Ouagadougou, Burkina Faso

WHO/APOC

20. Dr Uche Amazigo, Director, P.O. Box 549, Ouagadougou, Burkina Faso.
21. Dr Laurent Yaméogo, COORD, P.O. Box 549, Ouagadougou, Burkina Faso.
22. Dr Mounkaïla Noma, Chief, Elimination of Vector (CEV), P.O. Box 549, Ouagadougou, Burkina Faso.
23. Dr Lamissa Bangali, P.O. Box 549, Ouagadougou, Burkina Faso.
24. M. Honorat Zouré, Responsable chargé de la Biostistique, Informatique et Cartographie (BIM), Programme Africain de Lutte contre l'Onchocercose (APOC) 01 B.P. 549, Ouagadougou 01, Burkina Faso.
25. M. Saïdou N'Gadjaga, P.O. Box 549, Ouagadougou, Burkina Faso.
26. M. Issaka Niandou, P.O. Box 549, Ouagadougou, Burkina Faso.

ANNEX 3: LIST OF KEY DOCUMENTS

1. Recommendation of APOC External Evaluation team.

2. Report of External Evaluation Team, APOC, October 2005.

3. Memorandum for the African Programme for Onchocerciasis Control (APOC)- Phase II (2002-2007) and Phasing out period (2008-2010).

4. Current status of the implementation of APOC projects and its implementation on the duration of the Programme.

5. Defeating River Blindness in Africa – A vision for the future.

6. River blindness – Onchocerciasis and Ivermectin.

7. The future of Onchocerciasis control in Africa-context and reflections. A contribution to the "brainstorming session" on the future of Onchocerciasis control, 20-21 March, 2006.

8. Fact sheet-African Programme for Onchocerciasis control.

9. APOC funded projects.

10. Rapid epidemiological mapping of Onchocerciasis (REMO) in APOC countries.

11. African Programme for Onchocerciasis Control-Report of the External Evaluation, October 2005.

12. Kim A & Benton B (1995) Cost-Benefit Analysis of the Onchocerciasis Control Program (OCP). The World Bank, Washington, DC.

13. Dadzie, et al. (2003) "Final report of the Conference on the eradicability of Onchocerciasis". *Filaria Journal*. 2(2).

World Health Organization
Organisation Mondiale de la Santé
African Programme for
onchocerciasiste Control (APOC)
P.O Box 549
Ouagadougou Burkina Faso
Tél: +226-50 34 29 53/50 34 29 60
Télex: ONCHO 5241

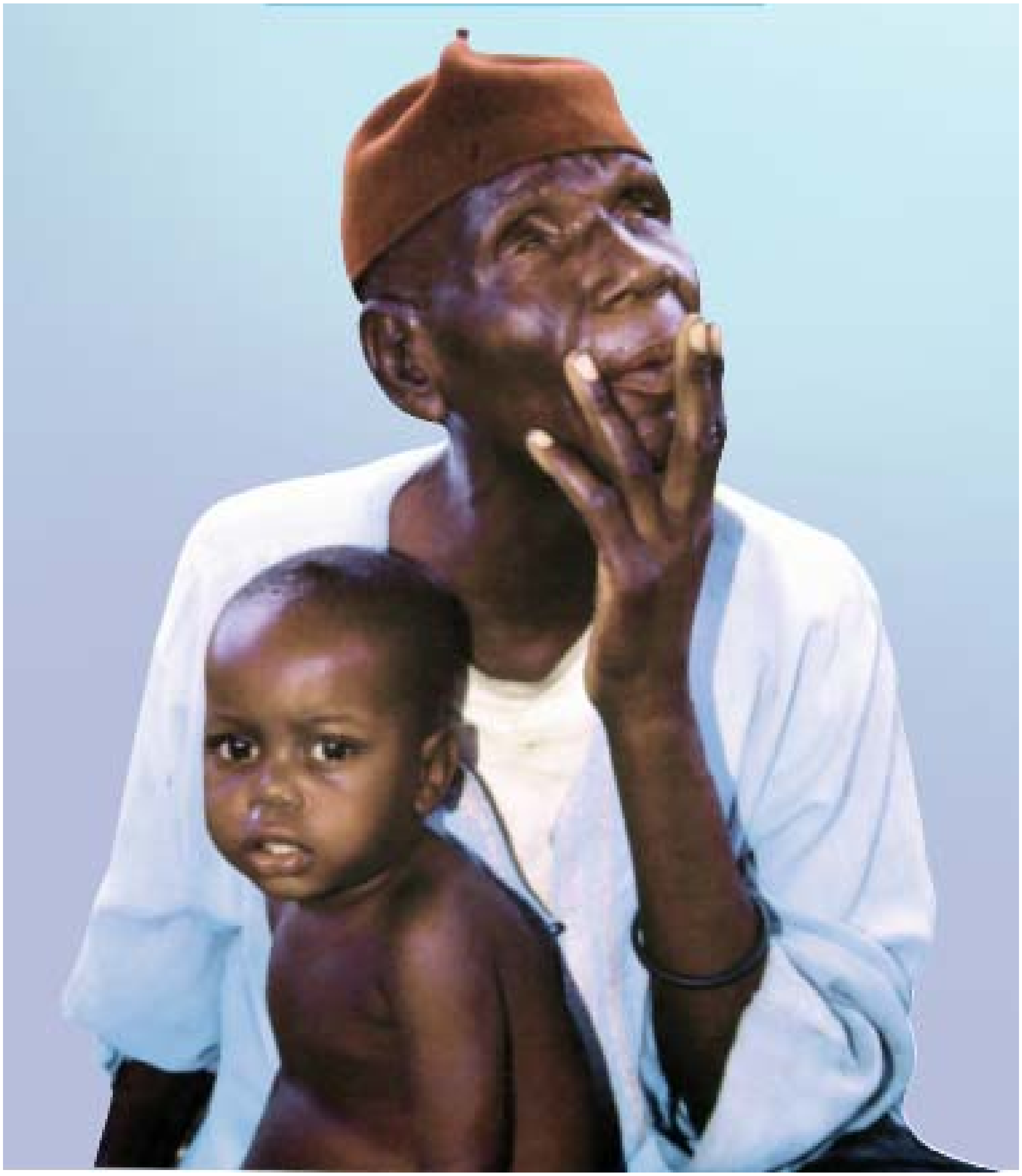

WHO/APOC/August 2006
ORIGINAL : ENGLISH
